# Anti-malarial drug use, appropriateness and associated factors among children under-five with febrile illnesses presenting to a tertiary health facility: a cross sectional study

**DOI:** 10.1186/s12936-023-04534-1

**Published:** 2023-03-21

**Authors:** Richard Nyeko, Felix Otim, Evelyn Miriam Obiya, Catherine Abala

**Affiliations:** 1 Department of Paediatrics and Child Health, Faculty of Medicine, Lira University, Lira, Uganda; 2 Department of Laboratory, St. Mary’s Hospital Lacor, Gulu, Uganda; 3 Department of Paediatrics and Child Health, St. Mary’s Hospital Lacor, Gulu, Uganda

**Keywords:** Anti-malarial, Febrile illnesses, Children, Appropriateness, Over-the-counter, Self-medication, Empiric prescription

## Abstract

**Background:**

Malaria is endemic in 95% of Uganda and constitutes the country’s most significant public health problem—being the leading cause of morbidity and mortality, especially among children under five years of age. The current national malaria treatment policy is to use artemisinin-based combination therapy (ACT) as first-line treatment, and recommends parasitological confirmation of malaria before therapy. Adherence to this policy, however, remains suboptimal, with the self-initiated home-based therapy being common—posing undue exposures to, and pressure on the current artemisinin-based combinations, with the danger of emergence of drug resistance. The study evaluated the anti-malarial use and its appropriateness among febrile children under five presenting to a tertiary health facility in northern Uganda in light of the current malaria treatment policy.

**Methods:**

This was a cross-sectional study in a tertiary health facility in northern Uganda between March and September 2021. Children aged 6–59 months with fever were selected using systematic random sampling. A pretested interviewer-administered questionnaire was used to collect clinical data from the caregivers. Data were analysed using SPSS version 23. Descriptive statistics and multiple logistic regression models were applied. P-value < 0.05 was considered for statistical significance.

**Results:**

Seventy-two (34.3%) of the 210 children with fever in this study used anti-malarials prior to the hospital visit, 29.2% (21/72) of which were on a self-medication basis, 22.2% (16/72) were empiric prescriptions—all of which inappropriate, and only 48.6% (35/72) were prescribed based on a parasitological diagnosis of malaria. The most commonly used anti-malarials were artemether-lumefantrine 60/72 (88.3%), while a lesser proportion of quinine 7/72 (9.7%), artesunate 3/72 (4.2%) and dihydroartemisinin-piperaquine 2/72 (2.8%) were used. The factors independently associated with anti-malarial use among the children with febrile illnesses were duration of fever (p = 0.001); level of the nearest facility (p = 0.027), distance from the nearest health facility (p = 0.025), and caregivers’ age (p = 0.038).

**Conclusions:**

Inappropriate use of anti-malarials for childhood febrile illnesses is prevalent in the study setting, facilitated by the ease of over-the-counter access, empiric prescription and use of leftover anti-malarials. This calls for a need to address communities’ health-seeking behaviour and the health providers’ practice alike.

## Background

Malaria remains among the top ten causes of death and an important public health problem in many African countries [1]. The World Health Organization (WHO) estimates indicate that there were 241 million cases of malaria worldwide and 627,000 deaths in 2020, mostly among young children in sub-Saharan Africa [2]. In Uganda, malaria is endemic in 95% of the country with stable transmission throughout the year, constituting the country’s most significant public health problem [3, 4]. It is the leading cause of morbidity and mortality, accounting for up to 40% of all outpatient visits at healthcare facilities, 20% of hospital admissions and 15% of inpatient deaths [3]. Children under 5 years of age bear the greatest burden of malaria, with the first one or two years of life, when most children experience their first malaria infection because of inadequate acquired immunity, being the most dangerous.

Prompt and accurate diagnosis is the cornerstone of effective malaria management and is fundamental to preventing morbidity and mortality while avoiding the unnecessary use of anti-malarial agents [5, 6]. However, in developing countries, Uganda inclusive, where malaria is endemic, fever in children is usually recognized by the caretakers as synonymous with malaria [7, 8], and over 50–75% of febrile illnesses are managed at home [9, 10]. Likewise, in these settings, clinical diagnosis using fever as the major symptom and empiric treatment of malaria, has historically been the most widely used approach by health practitioners [11, 12]. While this aligns with the early and prompt treatment of malaria aimed at reducing morbidity and mortality, especially in the under fives, these home-initiated and empiric treatments may often be inappropriate [13]. This approach has very low diagnostic accuracy given the fact that the symptom complex of malaria overlaps with those of many other tropical diseases [8, 11, 14].

To this effect, and in line with the WHO 2010 recommendations, Uganda’s current malaria treatment policy is to use artemisinin-based combination therapy (ACT) as first-line treatment, with parasitological confirmation of malaria before therapy by either malaria rapid diagnostic test (RDT) or microscopy [15–17]. This aims to; (i) minimize undue exposures and reduce drug pressure to the artemisinin-based combinations to slow the emergence of resistance to artemisinin and its partner drugs by encouraging more rational use of anti-malarials [18], and (ii) promote the diagnosis of other febrile illnesses in patients without malaria [19]. Recent pieces of evidence indicate the emergence, as well as local spread, of clinically artemisinin-resistant *Plasmodium falciparum* in Africa [20, 21], in the setting of a substantial increase in the volumes of artemisinin-based combinations purchased and distributed through private and retail sector channels [22]. Adherence to the treatment guidelines by both prescribers, drug shop outlets, pharmacists and patients alike is thus very crucial to the success of the current anti-malarial use policy [23].

Even though the parasitological diagnosis of malaria, especially with the advent of RDT, can be achieved at the community level [24, 25] and in private healthcare settings [26, 27], adherence to the test-and-treat guidelines remains wanting. For example, in one study in rural Uganda, poor adherence to the malaria treatment policy resulted in anti-malarial drug prescription in 30% of the patients with negative RDT test results, attributed to the fear of the consequences that can arise from severe malaria given the long distance to health facilities [4]. In the same study, children under five years were nearly 3 times more likely to receive anti-malarials relative to older patients, suggesting a disconnect between policy and practice. This is concerning for possible anti-malarial drug wastage and exposing patients to the dangers of inappropriate treatment, and the emergence of drug resistance [4]. While English and colleagues also argue in favour of presumptive treatment in the face of the wide availability of RDTs [28], evidence from a recent study in Uganda indicates that it is safe to withhold treatment for febrile smear-negative patients for all age groups [29]. The greatest threat to Sustainable Development Goal (SDG) 3 is the global growth in antimicrobial resistance, which is partly caused by irrational and improper use of the available antimicrobials, particularly in developing nations. Yet, in the majority of the contexts where it's thought to be a major problem, the scope and variables contributing to the inappropriate use of these antimicrobials are still not fully understood. This has to be looked into, and hence the rationale for this study.

This study aimed to evaluate anti-malarial drug use and its appropriateness among children under-five with febrile illnesses presenting to a tertiary health facility in northern Uganda in light of the current anti-malarial treatment policy and to determine the factors associated with these practices.

## Methods

### Study design and setting

This was a cross-sectional study design which used systematic random sampling to select participants and collect quantitative data over a period of seven months from March 2021 to September 2021. Children aged 6–59 months with documented fever at presentation were included in the study, while those with known chronic illnesses taking recommended routine anti-malarial prophylaxis, including children with sickle cell anaemia, were excluded. The study was conducted in the Paediatric Outpatient Department of St. Mary’s Hospital Lacor in northern Uganda, Gulu district, located approximately 330 km north of Kampala, Uganda's capital city. This is a private not-for-profit faith-based 482-bed capacity facility with a high patient load and a wide catchment area, receiving, on average, an annual outpatient attendance of children aged below 6 years of 22,142 [30].

### Study population and sample size estimation

The study population comprised children aged 6–59 months with fever presenting to the tertiary health facility during the study period. A sample size of 210 participants was estimated using the single population proportion formula of Kish [31], with a marginal error (D) of 5% and a standard normal value (Z) corresponding to 95% certainty (1.96).

### Study procedures

A pre-tested semi-structured questionnaire was used to collect socio-demographic data, pre-hospital medications, source of medicines, reasons for pre-hospital medication, and the child’s related symptoms. The questionnaire was translated and administered in the local language commonly spoken in the region/study setting. Pre-hospital exposures to anti-malarials among children presenting with fever were classified into two basic categories (a) inappropriate if the child was treated without parasitological confirmation, and (b) appropriate if treatment was based on parasitological confirmation of malaria infection.

### Outcome variables

The outcome variable was the proportion of febrile children with pre-hospital exposure to anti-malarials, while the predictor variables included the child’s and caregivers’ demographic characteristics, and the child’s related symptoms.

### Data management and statistical analysis

Data were analyzed using Statistical Package for Social Sciences (SPSS) software package (SPSS for Windows, Version 23.0. Chicago, SPSS Inc.). Descriptive statistics were used to summarize categorical variables as proportions and continuous variables as means and median. The Chi-square test or Fischer’s exact test (for categorical variables) and Student t-test (for continuous variables) with odds ratios and 95% confidence intervals were performed to determine the association between the predictor variables and pre-hospital exposures to anti-malarials. The multivariate logistic regression model with the backward stepwise method was used to assess the factors that independently predicted pre-hospital anti-malarial use and reported by an adjusted odds ratio (AOR) at a 95% confidence level. P-value < 0.05 was considered for statistical significance. Variables that were statistically significant at the bivariate level (p < 0.05) and those with p values < 0.2 were included in the multivariate model.

## Results

### Description of the study population

**Fig. 1:**
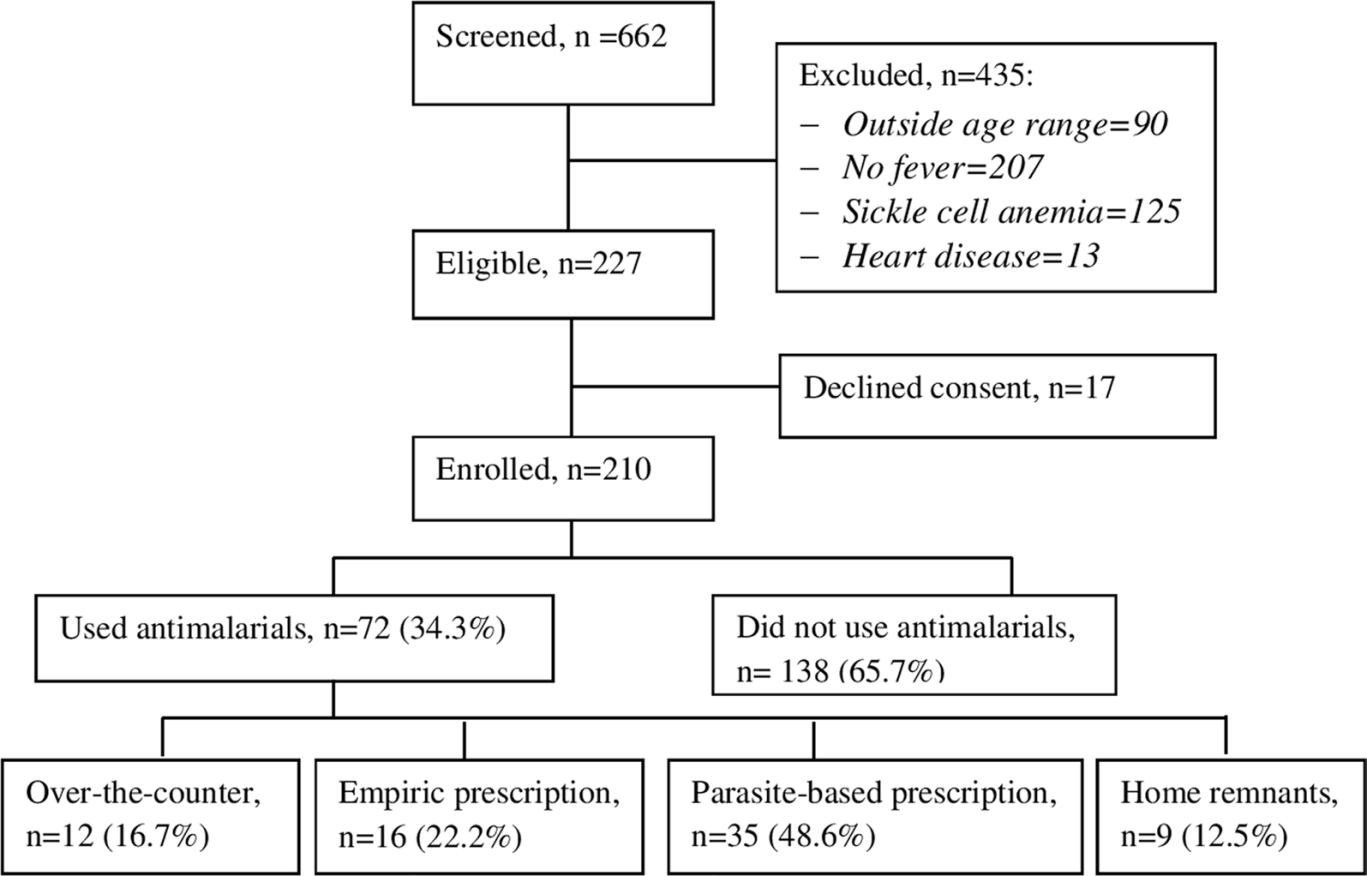
Study profile

During the study period, a total of 210 children under the age of 5 years who presented to the paediatric outpatient clinic had documented fever and were recruited into the study (Fig. 1).

The children had a median age of 22.0 months (IQR 14.0–39.5), and up to 82.4% (173/210) lived within 5 km or less from the nearest health facility; 56.2% (118/210) of the children were males. The majority of the children had a fever for 3 days or less (68.1%) and a negative malaria parasite test (73.3%). The majority of the caregivers were females, 91.9% (193/210) and the biological mothers of the children, 85.2% (179/210), while 31 (14.8%) were other relations. The mean age of the caregivers was 30.0 (SD 7.9 years), with a range of 18 to 60 years. One hundred and nine caregivers (51.9%) had primary or no education, while 101 (48.1%) had secondary or tertiary education (Table 1).

**Table 1:** Demographic characteristics of the study population

Variable			N	%
Child characteristics				
Age (months)	Median 22.0 (IQR 14.0–9.5)	Range (6–59)		
≤ 24			128	61
> 24–59			82	39
Sex				
Male			118	56.2
Female			92	43.8
Fever duration (days)				
≤ 3			143	68.1
> 3			67	31.9
Malaria parasitology				
Positive			49	23.3
Negative			154	73.3
Haemoglobin (Hb) g/dL	Mean 9.8 (SD 2.3)			
Distance nearest health facility				
≤ 5 km			173	82.4
> 5 km			37	17.6
Caregiver characteristics				
Sex				
Female			193	91.9
Male			17	8.1
Age (years)	Mean 30.0 (SD 7.9)	Range (18–60)		
≤ 30			135	64.3
>30			75	35.7
Relation				
Mother			179	85.2
Other			31	14.8
Education level				
≤ Primary			109	51.9
≥ Secondary			101	48.1
Children cared for				
1 child			52	24.8
> 1 child			158	75.2

### Clinical characteristics of the children presenting with fever

**Fig. 2:**
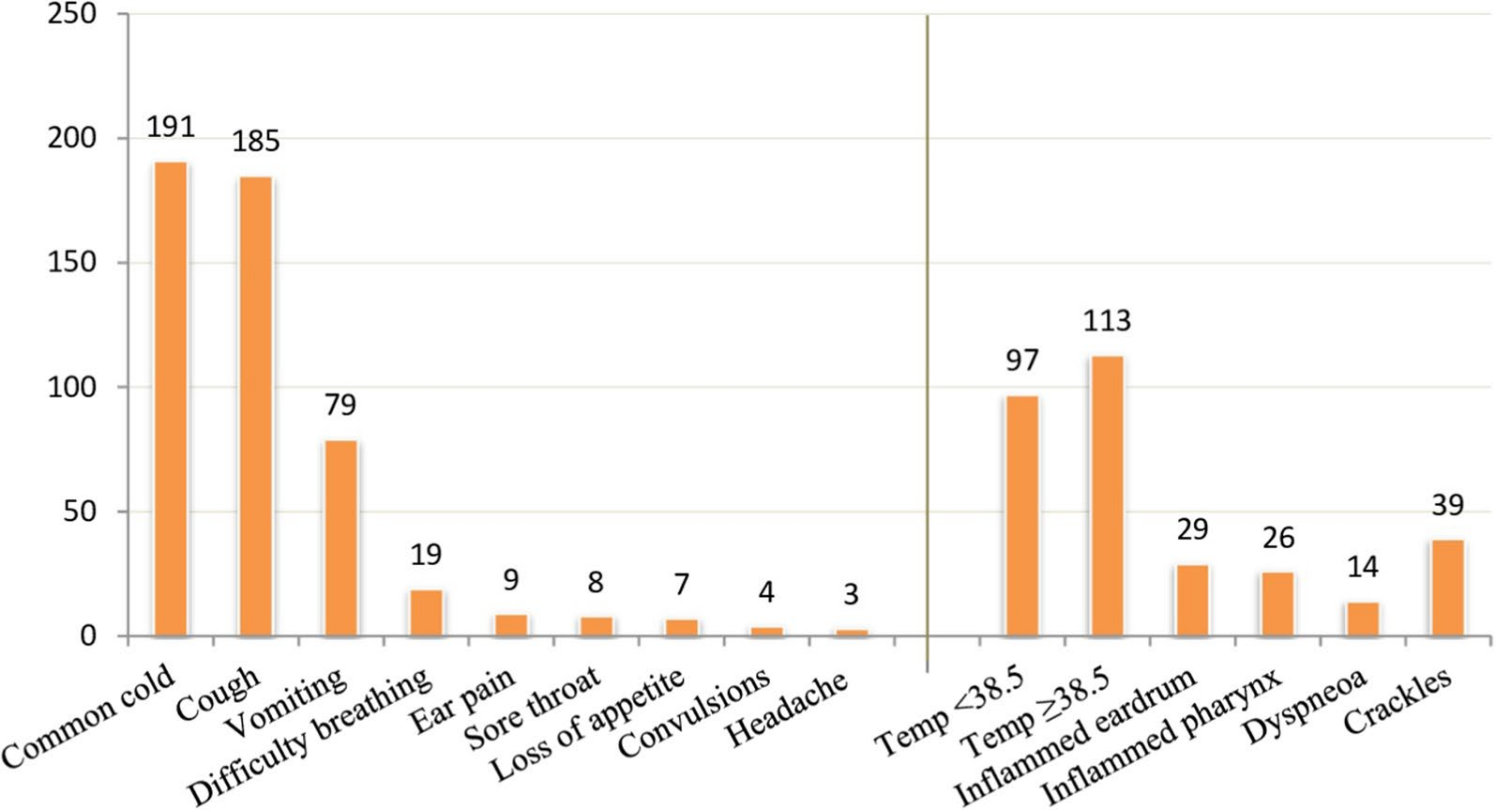
Associated symptoms among the children presenting with fever

The most common associated symptoms among children presenting with fever were common cold (rhinorrhea) 90.5% (191/210), cough 87.7% (185/210), and vomiting 37.6% (79/210) (Fig. 2). The majority of the children had a presenting temperature of ≥ 38.50C 53.8% (113/201). Common related clinical findings included lung crackles at 18.6% (39/210), inflamed eardrum at 13.8% (29/210), inflamed pharynx at 12.4% (26/210) and dyspnoea at 6.7% (14/210). The rest of the associated symptoms are shown in Fig. 2.

### Anti-malarial drug use among children under five presenting with fever at a tertiary health facility in northern Uganda

Seventy-two (34.3%) of the 210 children with febrile illnesses had received anti-malarials prior to presentation to the tertiary hospital. The most commonly used anti-malarial drug was artemether-lumefantrine 83.3% (60/72), followed by quinine 9.7% (7/72), artesunate 4.2% (3/72) and dihydroartemisinin-piperaquine 2.8% (2/72). The main sources of the anti-malarials were: over-the-counter purchases and leftovers at home, which represent self-medication and improper anti-malarial use; empiric prescriptions, which represent provider-related improper use; and parasitological-based prescriptions, which represent appropriate anti-malarial use (Table 2). As would be expected in children with febrile illnesses, the other medications used were paracetamol 69.0% (145/210) and antibiotics 39.5% (83/210).

**Table 2:** Anti-malarial drug use among children under five presenting with fever at a tertiary health facility in northern Uganda

Variable	N	%
Anti-malarial (n = 72)		
Artemether-lumefantrine	60	83.3
Quinine	7	9.7
Artesunate	3	4.2
Dihydroartemisinin-piperaquine	2	12.8
Other medications (n = 210)		
Paracetamol	145	69
Antibiotics	83	39.5
Others	7	3.3
Source of anti-malarial drugs (n = 72)		
Drug shops (over-the-counter)	12	16.7
Empiric prescription	16	22.2
Parasitological-based prescription	35	48.6
Remnants at home	9	12.5

### Factors associated with anti-malarial use among children under five with febrile illnesses presenting to a tertiary health facility in northern Uganda

Fever lasting 3 days or less was less likely to result in the use of anti-malarials for children with fever prior to hospital visits compared with fever lasting more than 3 days, and this remained statistically significant at multivariate analysis (p = 0.001, OR 0.31, 95% CI 0.16–0.62). Likewise, children whose nearest health facility was at the level of a hospital were less likely to be given anti-malarials prior to a hospital visit than those nearer lower-level facilities (p = 0.027, OR 0.39, 95% CI 0.17–0.90).

Residing within a distance of 5 km or less from the nearest health facility was also significantly independently associated with less likelihood of use of anti-malarials during febrile illnesses prior to hospital visits (p = 0.025, OR 0.41, 95% CI 0.18–0.89). While pre-hospital use of antibiotics was associated with the use of anti-malarial at bivariate analysis (p < 0.001, OR 3.31, 95% CI 1.83–5.99), this did not independently predict the use of anti-malarial at multivariate analysis (p = 0.070, OR 1.86, 95% CI 0.95–3.64) (Table 3).

**Table 3:** Child’s Demographic characteristics associated with pre-hospital anti-malarial use

Variable	Anti-malarial use		Bivariate		Multivariate^¥^	
	Yes	No	OR (95% CI)	*P*-value	aOR (95% CI)	*P*-value
	n = 72 (%)	n = 138 (%)				
Age (months)						
≤ 24	40 (31.1)	88 (68.8)	0.71 (0.40–1.27)	0.247	–	
> 24–59	32 (39.0)	50 (61.0)	1			
Sex						
Male	37 (31.4)	81 (68.6)	0.74 (0.42–1.32)	0.311	–	
Female	35 (38.0)	57 (62.0)	1			
Fever duration (days)						
≤ 3	32 (22.4)	111 (77.6)	0.20 (0.10–0.36)	< 0.001	0.31 (0.16–0.62)	0.001
> 3	40 (59.7)	27 (40.3)	1		1	
Temperature (°C)						
< 38.5	32 (33.0)	65 (67.0)	0.90 (0.51–1.59)	0.714	–	
≥ 38.5	40 (35.4)	73 (64.6)	1			
Cough						
Yes	67 (36.2)	118 (63.8)	2.27 (0.82–6.33)	0.109	0.88 (0.28–2.74)	0.821
No	5 (20.0)	20 (80.0)	1		1	
Vomiting						
Yes	28 (35.4)	51 (64.6)	1.09 (0.60–1.95)	0.784	–	
No	44 (33.6)	87 (66.4)	1			
Antibiotic use						
Yes	42 (50.6)	41 (49.4)	3.31 (1.83–5.99)	< 0.001	1.86 (0.95–3.64)	0.07
No	30 (23.6)	97 (76.4)	1		1	
Nearest HF						
Hospital	9 (14.5)	53 (85.5)	0.23 (0.11–0.50)	< 0.001	0.39 (0.17–0.90)	0.027
Health centre	63 (42.6)	85 (57.4)	1		1	
Distance						
≤ 5 km	52 (30.1)	121 (69.9)	0.37 (0.18–0.75)	0.005	0.41 (0.18–0.89)	0.025
> 5 km	20 (54.1)	17 (45.9)				

*aOR* adjusted Odds Ration;* ¥ method* backward stepwise

Regarding caregivers’ characteristics, caregivers aged ≤ 30 years and those caring for only one child were statistically significantly less likely to use anti-malarials in children with fever prior to hospital visits than their counterparts who were aged ˃30 years, p = 0.002 (OR 0.40; 95% CI 0.22–0.71) or caring for two or more children p = 0.003 (OR 0.32; 95% CI 0.14–0.69) respectively. However, only age remained independently predictive of prior anti-malarial use at multivariate analysis, p = 0.038 (OR 0.50; 95% CI 0.26–0.96) (Table 4).

**Table 4:** Caregivers’ Demographic characteristics associated with pre-hospital anti-malarial use

Variable	Anti-malarial use		Bivariate		Multivariate^¥^	
	Yes	No	OR (95% CI)	*P*-value	aOR (95% CI)	*P*-value
	n = 72 (%)	n = 138 (%)				
Age (years)						
≤ 30	36 (26.7)	99 (73.3)	0.40 (0.22–0.71)	0.002	0.50 (0.26–0.96)	0.038
>30	36 (48.0)	39 (52.0)	1		1	
Sex						
Female	65 (33.7)	128 (66.3)	0.73 (0.26–1.99)	0.532	–	
Male	7 (41.2)	10 (58.8)	1			
Education level						
≤ Primary	44 (40.4)	65 (59.6)	1.77 (1.00–3.15)	0.054	1.66 (0.90–3.07)	0.106
≥ Secondary	28 (27.7)	73 (72.3)	1		1	
Relation						
Mother	62 (34.6)	117 (65.4)	1.11 (0.49–2.51)	0.797	–	
Other	10 (32.3)	21 (67.7)	1			
#Children cared for						
1 child	9 (17.3)	34 (82.7)	0.32 (0.14–0.69)	0.003	0.44 (0.18–1.06)	0.067
≥ 2 children	63 (39.9)	95 (60.1)	1		1	

*aOR* adjusted Odds Ration; *¥ method* backward stepwise; *#* number of

## Discussion

In low- and middle-income countries, caregivers often initiate treatment at home before a visit to the health facility, including the administration of artemisinin-based anti-malarials, largely on a self-medication basis, contrary to the current shift in malaria treatment policy from presumptive treatment to mandatory testing of all suspected malaria cases. This poses a potential threat to the stewardship of, and the emergence of resistance to artemisinin-based therapy. The current facility-based study investigated anti-malarial drug use and its appropriateness among children under-five with febrile illnesses presenting to a tertiary health facility in northern Uganda in light of the current anti-malarial treatment policy and to determine the factors associated with these practices. It highlights the challenges of inappropriate anti-malarial use among children in a resource-limited context.

### Prevalence and characteristics of pre-hospital anti-malarial use among children under five with febrile illnesses presenting to a tertiary health facility

This study found that 34.3% of children under five presenting with fever to a tertiary-level health facility in northern Uganda used anti-malarials, only 48.6% of which conformed to the prevailing national guidelines for the management of malaria. However, evidence from a recent antibiotic study indicating a low validity of caregivers' reports on the prior intake of antibacterials by their children (14.4% reported vs. 63.7% antibacterial detection in blood and urine samples) suggests that the 43.3% rate of anti-malarial use in the current study may be an underestimate [32]. The rate of pre-hospital exposure to anti-malarials in this study, however, may not be surprising given the endemicity of malaria in Uganda – being the most common cause of morbidity, especially in children under five, and the fact that these medicines are readily available as over-the-counter medication. The malaria treatment policy and the integrated community case management (iCCM) strategy [33] recommend prompt initiation of effective and appropriate treatment within 24 h of onset of illness—making this an important finding.

Nevertheless, more crucially, this study’s findings demonstrate how ineffectively the iCCM strategy was being implemented. This strategy proposes therapy based on parasitological confirmation of suspected cases of malaria by performing a quick diagnostic test [33]. The fact that a higher percentage of anti-malarials were obtained from over-the-counter purchases, leftovers at home, and empiric prescriptions—all of which represent inappropriate anti-malarial use resulting from self-medication and provider-related sources—before a visit to the tertiary hospital illustrates this. This finding resonates with an earlier report in Uganda [13] and attests to a finding that in Uganda, pharmacies, drug shops, and clinics dispense whatever medicines the client requests [34]. The high rate of over-the-counter purchase and use of remnants at home could present enormous challenges, including delayed healthcare-seeking, exacerbation or masked symptoms, or the development of resistance to the current anti-malarials. Recent evidence does point to the emergence of artemisinin partial resistance in the WHO African Region [2]. The use of home remnants is also concerning because of the risk of inappropriate dosing, expiry, loss of potency and increased toxicity from unideal storage conditions [35], as well as administering the wrong medication for a wrong diagnosis. It’s also an indicator of poor compliance with, and adherence to, medication dosages – a preposition supported by a finding by Cohen et al*.* [36], where 59.6% of the nonadherent patients had more than a full day of treatment remaining.

### Factors associated with pre-hospital anti-malarial use among children under five with febrile illnesses presenting to a tertiary health facility

The duration of fever was a statistically significant predictor of anti-malarial use among febrile children. Children who had a fever for 3 days or less had a 69% reduced likelihood of using anti-malarials prior to presenting to the tertiary health facility compared to their counterparts with fever lasting more than 3 days. This finding corroborates an earlier report from Uganda where children who received pre-hospital medications had a twofold increased likelihood to present after three days of fever than their counterparts who did not receive pre-hospital medications [13]. This is an important finding because it seems to suggest the negative effect of pre-hospital and home medications on the timeliness of seeking appropriate care from a health facility by delaying presentation to the health facility. This proposition is supported by a finding by Nshakira et al*.* [37] in a community study where patients who received pre-hospital medications had a 1.3 days delay to present to the hospital compared to those who did not receive any medication. This could have far-reaching implications since delay to seek appropriate care while receiving inappropriate medication and/or dosages may result in disease progression, potential untoward complications and prolonged hospital stay, and disability, among other consequences.

Residing within a distance of 5 km or less from the nearest health facility was associated with a 59% reduced likelihood of using anti-malarials prior to a hospital visit. This finding resonates with the well-known fact that the distance from a health facility is an important determinant of access to healthcare—a reason advanced by the respondents for choosing to give treatment from home and speaks to the importance of improving access to healthcare services in low-income countries. The effect of distance on the use of anti-malarials was brought to the fore in a study in rural Uganda, where prescribers attributed poor adherence to the malaria treatment policy to the fear of the consequences that can arise from severe malaria given the long distance to health facilities [4]. However, in their study in Uganda, Tumukunde et al*.* [13] did not find a significant correlation between distance and the use of anti-malaria medicines prior to presentation in the hospital. This could possibly be explained by the differences in the contexts of the two studies—an upcountry setting in the current study and a metropolitan capital city in the other study where availability and access to healthcare services are quite contrasting.

While the distance from the nearest health facility is an important determinant of pre-hospital medications, the level of the health facility that is most easily accessible to the respondents also seems to play an important role in the decision towards the management of childhood febrile illnesses. Children residing nearer to a facility which is at the level of a hospital had 61% less likelihood of receiving anti-malarial treatment prior to the visit to a tertiary care facility compared to those whose nearest health facility was at the level of a health centre and lower. It could be argued that this could be reflective of the level of confidence the community have in these lower public health facilities to manage their children’s conditions. This study did not find a statistically significant association between the child’s age and anti-malarial use, contrary to a report by Elechi et al*.* [4] where children under five years were nearly 3 times more likely to receive anti-malarials relative to older patients resistance [4]—which is concerning for exposing many the under fives to the dangers of inappropriate treatment.

Another important finding from this study relates to the influence of caregivers’ age on the use of anti-malarials among children with fever. Children whose caregivers were aged 30 years and below had a 50% reduced likelihood of exposure to anti-malarials prior to a hospital visit compared to those whose caregivers were aged above 30 years. This finding corroborates with a report by Al Rasheed et al*.* [38] in the Kingdom Republic of Saudi Arabia indicating a higher probability of self-prescription among respondents older than 30 years compared to those aged 18–30 years, probably attributed to previous experiences that develop over time.

Our findings could have been limited by recall and information bias, and the low reliability of the reported use of antimicrobials by patients. However, this was minimized by the use of a simple and easy-to-understand questionnaire administered confidentially. The single-centre nature of the study could have also affected the external validity of the study findings since a multicentre study could not be carried out because of limited resources. The choice of the study site was, however, meant to circumvent this limitation since this is one of the largest tertiary health facilities in the region with a large catchment area, attracting patients from all over the region.

## Conclusions

The pre-hospital exposure to anti-malarials in the study setting is high, and largely inappropriate, driven by the ease of access through drug shops, pharmacies, and clinics and the use of remnant medications from previous illnesses. Adherence to the current malaria treatment recommendations is therefore suboptimal. Factors determining pre-hospital anti-malarial exposures include the duration of fever, level of the nearest health facility, distance from the nearest health facility, and caregivers’ age. There is a need for adequate training and sensitization of the community and health providers alike with the relevant information to promote effective uptake of, and compliance with, the current malaria treatment policies.

## Data Availability

The datasets used and/or analysed during the current study are available from the corresponding author upon reasonable request.

## Abbreviations

WHO: World Health Organization
ACT: Artemisinin-based Combination Therapy
RDT: Malaria Rapid Diagnostic Test
SPSS: Statistical Package for Social Sciences
IQR: Inter-Quartile Range
SD: Standard Deviation
